# Effects of Social Mobility and Stringency Measures on the COVID-19 Outcomes: Evidence From the United States

**DOI:** 10.3389/fpubh.2021.779501

**Published:** 2021-11-04

**Authors:** Jianmin Sun, Keh Kwek, Min Li, Hongzhou Shen

**Affiliations:** ^1^School of Management, Nanjing University of Posts and Telecommunications, Nanjing, China; ^2^School of Management, Qingdao City University, Qingdao, China; ^3^School of Economics, Tianjin University of Commerce, Tianjin, China

**Keywords:** COVID-19 outcomes, social mobility, stringency measures, the United States economy, ordinary least squares, machine learning estimator

## Abstract

This paper examines the effects of stringency measures (provided by the Oxford Coronavirus Government Response Tracker) and total time spent away from home (provided by the Google COVID-19 Community Mobility Reports) on the COVID-19 outcomes (measured by total COVID-19 cases and total deaths related to the COVID-19) in the United States. The paper focuses on the daily data from March 11, 2020 to August 13, 2021. The ordinary least squares and the machine learning estimators show that stringency measures are negatively related to the COVID-19 outcomes. A higher time spent away from home is positively associated with the COVID-19 outcomes. The paper also discusses the potential economic implications for the United States.

## Introduction

The Coronavirus disease 2019 (COVID-19) pandemic started in late 2019 and spread to each country within a few months. The pandemic has been one of the leading disasters in modern history and has negatively affected various economic and social indicators (1, 2). The COVID-19 pandemic started as a health crisis, but it also significantly damaged the world economy (3). Meanwhile, the pandemic has been the priority of policymakers in leading economies because the COVID-19 virus is more lethal than the common flu (4). Therefore, policymakers have implemented various implications to minimize social mobility, such as social distancing measures and mandatory lockdowns (5). These implications have negatively affected economic performance (6, 7). At this stage, it is important to understand whether these mandatory lockdowns to minimize social mobility and stringency measures are beneficial to reduce the COVID-19 outcomes, which are usually measured by the total COVID-19 cases and total deaths related to the COVID-19. For this purpose, this paper aims to analyze the effects of stringency measures and social mobility (total time spent away from home) on the COVID-19 outcomes (measured by total cases and total deaths related to the COVID-19) in the United States.

There are previous papers to examine the determinants of the COVID-19 outcomes. For instance, Khan et al. (8) observe that higher health expenditures and healthcare capacity decrease the COVID-19 case-fatality ratios. Countries with an older population experience higher deaths related to the COVID-19 relative to their COVID-19 cases. Haldar and Sethi (9) observe that demography and government interventions are the leading drivers of the COVID-19 outcomes. Interestingly, economic indicators, i.e., the per capita income and the human development index, have no significant effects on the COVID-19 outcomes. Allel et al. (10) also conclude that government policies are the main drivers of the COVID-19 outcomes. Sorci et al. (11) find that demographics (measured by the population over 70 relatives to the total population), economic development (measured by the per capita gross domestic product), and institutional quality (measured by democracy index) are the main determinants of the COVID-19 outcomes. Martinez-Valle (12) examines the effectiveness of governments' policy implications on the COVID-19 pandemic in Argentina, Brazil, Chile, Colombia, Mexico, and Peru. The author uses the stringency index and Google social mobility indicator and finds that stringency measures lead to lower mortality rates in all of these countries.

On the other hand, Moosa and Khatatbeh (13) demonstrate that demographics (measured by the age structure of the population) and social networks (measured by the population density) are the significant drivers of the COVID-19 case-fatality ratio. Daw (14) indicates that the armed conflicts are the main drivers of the COVID-19 outcomes in three case countries: Libya, Syria, and Yemen. Similarly, Zhai et al. (15) focus on the cross-sectional data in 120 countries and show that internal and external conflicts are the main determinants of the COVID-19 pandemic.

To the best of our knowledge, this paper is the first to examine the effects of total time spent away from home and stringency measures on the COVID-19 outcomes (measured by total cases and total deaths related to the COVID-19) in the United States. For this purpose, we consider a large time series spanning the daily data from March 11, 2020 to August 13, 2021. In addition, we utilize the ordinary least squares (OLS) and the kernel-based regularized least squares (KRLS) estimation techniques. According to the empirical findings, stringency measures are negatively related to the COVID-19 outcomes. Moreover, a higher time spent away from home is positively associated with the COVID-19 outcomes.

The remainder of the study is organized as follows. Section Model and Data explains the empirical model and the dataset in the empirical examination. Section Empirical Findings discusses the empirical findings, and section Conclusion provides the conclusion of the paper.

## Model and Data

### Empirical Model

This paper estimates the following model to examine the effects of social mobility (total time spent away from home) and stringency measures on the COVID-19 outcomes.


(1) $$\begin{array}{l} COVID_{CASES}=\alpha_{0}+\alpha_{1}MOB_{t}+\alpha_{2}STR_{t}+\varepsilon_{it} \end{array}$$

(2) $$\begin{array}{l} COVID_{DEATH}=\beta_{0}+\beta_{1}MOB_{t}+\beta_{2}STR_{t}+\varepsilon_{it} \end{array}$$

In Equations (1) and (2), *COVID*_*CASES*_ is the daily total COVID-19 cases. *COVID*_*DEATH*_ is the total daily deaths related to the COVID-19. *MOB*_*t*_ is the social mobility, and it is daily the total time spent away from home. *STR*_*t*_ is the stringency measures. ε_*it*_represents the error terms.

We expect that α_1_ > 0 and β_1_ > 0 as the social mobility should increase the COVID-19 outcomes. In addition, α_2_ <0 and β_2_ <0 as the stringency measures should decrease the COVID-19 outcomes. Following Zhai et al. (15), we estimate these models using the OLS and the machine learning estimators, the so-called KRLS, introduced by Hainmueller and Hazlett (16).

### Data

This paper focuses on the daily data from March 11, 2020 to August 13, 2021, in the United States. We have 521 observations for each indicator. The dependent variables are the COVID-19 outcomes, and two indicators are used: (1) Total COVID-19 cases in the logarithmic form and (2) Total deaths related to the COVID-19 in the logarithmic form. The related data are accessed from the Cross-country Database of COVID-19 Testing dataset provided by Hasell et al. (17).

We focus on two explanatory variables. First is the total time spent away from home to capture social mobility, and it is available in the Google COVID-19 Community Mobility Reports. We download the social mobility data from the website (https:/tracktherecovery.org/), introduced by Chetty et al. (18). We consider the relative change of the average time spent outside the residential locations, indexed from January 3, 2020 to February 6, 2020. The second explanatory variable is the stringency measures, available in the Oxford Coronavirus Government Response Tracker. We obtain stringency measures data from the website (https://www.bsg.ox.ac.uk/research/research-projects/covid-19-government-response-tracker), introduced by Hale et al. (19). This index is a composite measure based on nine response indicators: school closures, workplace closures, cancel public events, restrictions on gatherings, public transport closures, public information campaigns, stay-at-home restrictions on internal movement, and international travel controls. The index is rescaled to vary from 0 to 100, and a higher index level indicates a higher stringency measure.

Table 1 provides brief measures of descriptive statistics, including the average values, the standard deviations, the minimum values, and the maximum values of each indicator.

**Table 1:** Descriptive statistics.

**Indicator**	**Definition**	**References**	**Mean**	**Std. dev**.	**Min**.	**Max**.	**Obs**.
The COVID-19 outcome: total cases	Natural log	Hasell et al. (17 )	15.88	1.760	7.044	17.41	521
The COVID-19 outcome: total deaths	Natural log	Hasell et al. (17)	12.21	1.541	3.496	13.33	521
Social mobility: total time spent away from home	Per cent change	Chetty et al. (18)	−0.101	0.047	−0.237	0.006	521
Stringency measures	Index from 0 to 100	Hale et al. (19)	64.46	8.549	21.76	75.46	521

Table 2 reports a pairwise correlation matrix among the variables in the dataset. We observe that the correlation between the COVID-19 cases and the COVID-19 related deaths is 0.97. The correlation between social mobility and the COVID-19 cases is 0.23, and the COVID-19 related deaths are 0.14. Moreover, the correlation between the stringency index and the COVID-19 cases is −0.44, and the COVID-19 related deaths are −0.36. Finally, the correlation between social mobility and the stringency index is found as −0.76. The correlations among the indicators are in line with the theoretical expectations.

**Table 2:** Correlation matrix.

**Indicator**	**The log of total cases**	**The log of total deaths**	**Social mobility**	**Stringency**
**The log of total cases**	1	–	–	–
**The log of total deaths**	0.973	1	–	–
**Social mobility**	0.233	0.136	1	–
**Stringency**	−0.441	−0.363	−0.759	1

## Empirical Findings

### Total COVID-19 Cases

Table 3 provides the results of the COVID-19 outcomes, measured by the log of total cases.

**Table 3:** Results of the COVID-19 outcomes: the log of total cases.

**Indicator**	**OLS (1)**	**OLS (2)**	**OLS (3)**	**KRLS (4)**	**KRLS (5)**	**KRLS (6)**
Social mobility: total time spent away from home	16.27*** (2.173)	–	22.99*** (2.323)	11.91*** (1.749)	–	9.731*** (3.581)
Stringency measures	–	−0.048** (0.021)	−0.049* (0.026)	–	−0.086*** (0.016)	−0.138*** (0.018)
R-squared	0.1947	0.0545	0.2191	0.5971	0.4599	0.6432
Observations	521	521	521	521	521	521

*The dependent variable is the log of total cases, and the constant term is included. The robust standard errors are in the parentheses.*

***p < 0.01, **p < 0.05, and *p < 0.10.

Column (1) provides the results of the OLS estimations for social mobility, measured by total time spent away from home. The findings show that social mobility increases the log of total COVID-19 cases in the United States. The coefficient is statistically significant at the 1% level. Column (2) reports the findings of the OLS estimations for stringency measures. The result indicates that stringency measures reduce the log of total COVID-19 cases in the United States. The coefficient is statistically significant at the 5% level. Column (3) provides the findings of the OLS estimations for both social mobility and stringency measures. The findings indicate that social mobility increases the log of total COVID-19 cases and stringency measures reduce total COVID-19 cases in the United States. The coefficients are statistically significant.

Furthermore, Column (4) reports the findings of the KRLS estimations for social mobility, measured by total time spent away from home. The result indicates that social mobility increases the log of total COVID-19 cases. The coefficient is statistically significant at the 1% level. Column (5) provides the results of the KRLS estimations for stringency measures. The finding shows that stringency measures decrease the log of total COVID-19 cases. The coefficient is statistically significant at the 1% level. Column (6) reports the findings of the KRLS estimations for both social mobility and stringency measures. The results show that social mobility increases the log of total COVID-19 cases and stringency measures reduce total COVID-19 cases in the United States. The coefficients are statistically significant at the 1% level. The KRLS estimations have higher R-squared values than the OLS estimations, and thus, they have higher explanatory power than the OLS estimations. In short, the KRLS estimations can be seen as the benchmark results.

### Total COVID-19 Related Total Deaths

Table 4 reports the results of the COVID-19 outcomes, measured by the log of total deaths.

**Table 4:** Results of the COVID-19 outcomes: the log of total deaths.

**Indicator**	**OLS (1)**	**OLS (2)**	**OLS (3)**	**KRLS (4)**	**KRLS (5)**	**KRLS (6)**
Social mobility: total time spent away from home	11.73*** (2.128)	–	19.81*** (2.248)	2.918* (1.617)	–	6.455* (3.355)
Stringency measures	–	−0.024*** (0.009)	−0.059** (0.024)	–	−0.067*** (0.014)	−0.082*** (0.017)
R-squared	0.1319	0.1186	0.1778	0.5662	0.4611	0.6432
Observations	521	521	521	521	521	521

*The dependent variable is the log of total deaths, and the constant term is included. The robust standard errors are in the parentheses.*

*** p < 0.01, **p < 0.05, and *p < 0.10.

Column (1) reports the findings of the OLS estimations for total time spent away from home. The results indicate that social mobility increases the log of total deaths in the United States, and the coefficient is statistically significant at the 1% level. Column (2) provides the findings of the OLS estimations for stringency measures. The finding shows that stringency measures decrease the log of total deaths in the United States, and the coefficient is statistically significant at the 1% level. Column (3) reports the results of the OLS estimations for both social mobility and stringency measures. The results indicate that social mobility increases total deaths, and stringency measures decrease the total deaths related to the COVID-19 in the United States. The coefficients are statistically significant at the 5% level at least.

Column (4) reports the results of the KRLS estimations for total time spent away from home. The findings show that social mobility increases the log of total deaths. The coefficient is statistically significant at the 10% level. Column (5) provides the findings of the KRLS estimations for stringency measures. The results indicate that stringency measures reduce the log of total deaths. The coefficient is statistically significant at the 1% level. Column (6) reports the results of the KRLS estimations for both social mobility and stringency measures. The results show that social mobility increases the log of total COVID-19 related deaths, and stringency measures decrease it. The coefficients are statistically significant at the 10% level at least. Again, the KRLS estimations provide higher R-squared values than the OLS estimations. The KRLS estimations have higher explanatory power than the OLS estimations. In short, the KRLS estimations are the benchmark findings.

Overall, we observe that social mobility increases the log of total cases and total deaths in the United States. In addition, stringency measures decrease the log of total cases and the log of total deaths.

## Conclusions

The COVID-19 pandemic has continued for almost one and a half years, and the pandemic pattern is still difficult to predict. Most countries have increased the vaccination rate, but the stringency measures are still leading implications to slow down the spread of the virus, especially new variants. This paper uses the effects of stringency measures provided by the Oxford Coronavirus Government Response Tracker. We define this measure as the de jure limitations due to the COVID-19. We also consider the total time spent away from home, provided by the Google COVID-19 Community Mobility Reports. We define this measure as the de facto limitations due to the COVID-19. We examine the effects of both measures on the COVID-19 outcomes, measured by total cases and total deaths related to the COVID-19, in the United States. We use the daily data from March 11, 2020 to August 13, 2021. The results from the OLS and the KRLS estimators indicate that stringency measures negatively affect the COVID-19 outcomes. A higher time spent away from home is positively associated with the COVID-19 outcomes.

Our results show that stringency measures and social distancing are the main determinants of the COVID-19 outcomes in the United States. However, stringency measures can decrease macroeconomic activity; and therefore, they can negatively affect household consumption, small business activities, and employment. Besides, social distancing can decrease the level of social networks and can increase psychological problems at the individual level. Therefore, policymakers should determine the optimal limitations to balance stringency and the COVID-19 related deaths. This issue is a dynamic process due to the randomness of the pandemic with new variants; therefore, policymakers need active monitoring of the pattern of the pandemic. Testing and other data collection procedures are vital to determine the optimal limitations.

Finally, our paper obtains empirical evidence, limited to the United States, and future papers can focus on other advanced economies. Findings from developing countries can also be important, given that most of these countries have limited access to effective vaccines. At this stage, Brazil, India, and Russia are the leading candidates investigate the effects of stringency measures and total time spent away from home on the COVID-19 outcomes.

## Data Availability Statement

Publicly available datasets were analyzed in this study. This data can be found at: https:/tracktherecovery.org; https://www.bsg.ox.ac.uk/research/research-projects/covid-19-government-response-tracker.

## Author Contributions

JS: writing manuscript and methodology. KK: writing manuscript and data collection. ML: writing manuscript and conceptualization. HS: writing manuscript and validation. All authors contributed to the article and approved the submitted version.

## Funding

The authors acknowledge the financial support from the National Natural Science Foundation of China (Grant no: 71974102) and from the Philosophy & Social Science Fund of Tianjin City, China (Grant no: TJYJ20-012).

## Conflict of Interest

The authors declare that the research was conducted in the absence of any commercial or financial relationships that could be construed as a potential conflict of interest.

## Publisher's Note

All claims expressed in this article are solely those of the authors and do not necessarily represent those of their affiliated organizations, or those of the publisher, the editors and the reviewers. Any product that may be evaluated in this article, or claim that may be made by its manufacturer, is not guaranteed or endorsed by the publisher.
